# Novel and emerging therapies for B cell lymphoma

**DOI:** 10.1186/s13045-019-0752-3

**Published:** 2019-07-25

**Authors:** Sabarish Ayyappan, Kami Maddocks

**Affiliations:** Division of Hematology, Department of Internal Medicine, Arthur G. James Comprehensive Cancer Center, The Ohio State University Wexner Medical Center, 320 W 10th Street, A342 Starling Loving Hall, Columbus, Ohio 43210 USA

**Keywords:** Lymphoma, First in human study, Immunotherapy, Cellular therapy

## Abstract

Lymphomas are a heterogeneous group of lymphoproliferative disorders, with unique clinical and biological characteristics that exhibit variable response to therapy. Advances in chemo-immunotherapy have improved outcomes in a number of lymphoma subtypes; however, the prognosis for many patients with relapsed and refractory disease remains poor. Novel therapies including several small molecule inhibitors and chimeric antigen receptor T cells have been approved for the treatment of different lymphoma subtypes at relapse, changing the therapy landscape and further improving survival in many of these diseases. This has led to a focus on the development of new cellular therapy, antibody-based therapy, and small molecule inhibitors for relapsed and refractory disease that offer an alternative approach to cytotoxic chemotherapy. We will review these promising novel therapies and discuss their safety and efficacy in first in human studies.

## Background

Lymphomas, including Hodgkin (HL) and non-Hodgkin lymphoma (NHL), are a heterogeneous group of B cell-derived lymphoproliferative malignancies with varying patterns of clinical behavior and treatment responses. Given the progress in the understanding of different disease biology, the discovery of newer treatments has resulted in increased survival. More effective chemotherapy regimens, newer monoclonal antibodies, radio-immunotherapy, and adoptive T cell therapy have improved the management of lymphomas. However, there are patients who relapse and are refractory to conventional therapy options requiring novel approaches. Herein, we present some early results of first in human studies utilizing promising new approaches to relapsed and refractory (r/r) lymphomas.

## Immunotherapy

Recent advances in cancer immunotherapy have improved outcomes in advanced malignancies including lymphomas [1–3]. In healthy individuals, the host immune system plays a central role in the diagnosis and prevention of cancer through identifying self and foreign antigens and malignant cell elimination [4]. However, malignancies evade the immune system through alteration of surface antigen expression and T cell exhaustion [5]. Earliest success with immune modulation for lymphoma management has been demonstrated with allogeneic stem cell transplantation through graft versus lymphoma effect, which has been shown to be effective in various histologies of NHL. Novel effective immune modulation can be directed through adoptive cellular therapy and immune cell-targeted monoclonal antibodies.

### Adoptive cellular therapy

Adoptive cellular therapy is a form of immunotherapy that involves ex vivo manipulation of autologous T cells followed by reinfusion which produces an immune-mediated tumor response. Early work in this field involved the discovery of tumor infiltrating lymphocytes (TIL), a subset of T lymphocytes targeting tumor-specific antigens. Tumor-specific antigens are necessary for tumor recognition by T cells and activation for tumor killing. T cell receptors (TCRs) are expressed on the surface of T cells and play a central role in the function of the adaptive immune system. TCRs can be engineered with epitope-specific activity for tumor recognition, T cell activation, and avoiding auto-immunity [6]. However, their function is limited by their ability to identify only short peptides and poor recognition of modifiers including glycosylation and, thus, can fail to identify multiple tumor antigens [6]. Antibody-derived recognition is not hampered by the peptide length and does not require antigens to be presented along with major histocompatibility complex (MHC) molecules. The chimeric antigen receptor (CAR) therapy has been designed by combining the antibody derived extracellular antigen-detecting domain with an intracellular domain providing TCR signaling to activate T cells.

#### CAR T cell therapy

CAR T cell therapies are engineered from autologous T cells by genetic modification to express a CAR that consists of a transmembrane protein with an extracellular antigen recognition domain to identify cancer cells, a transmembrane hinge, and an intracellular signaling domain for T cell activation. The autologous T cells from a patient are modified to express the chimeric protein, expanded in vivo, and reinfused into the patient. CAR T cells can recognize the tumor antigen, independent of the major histocompatibility complex and activate T cells leading to tumor cell death. Current CAR T cells utilize co-stimulatory molecules like CD-28, 4-1BB for the T cell proliferation and survival, producing a persistent antitumor effect. Recently, the U.S. Food and Drug Administration (FDA) approved CAR T cell therapies targeting CD-19 as the tumor antigen: tisagenlecleucel for recurrent pediatric acute lymphoblastic leukemia (ALL) [7] and r/r large B cell lymphoma [8, 9] and axicabtagene ciloleucel for r/r large B cell lymphoma [10, 11]. Lisocabtagene maraleucel is another CAR T cell product targeting the CD-19 antigen that has breakthrough designation from the FDA and has shown promising results in early trials [12]. Table 1 compares the properties, efficacy, and safety data from early phase trials for the three CAR T cell therapies in lymphoma.

**Table 1:** CAR T cell therapy in lymphoma

CAR T cell product	Axicabtagene ciloleucel (Yescarta)	Tisagenlecleucel (Kymriah)	Lisocabtagene maraleucel
Costimulation domain	CD-28	4-1BB	4-1BB
Vector	Retrovirus	Lentivirus	Lentivirus
Conditioning regimen	Fludarabine, cyclophosphamide	Fludarabine, cyclophosphamide, or bendamustine	Fludarabine, cyclophosphamide
Pivotal trial	ZUMA-1 (*N* = 108)	JULIET (*N* = 111)	TRANSCEND-NHL-001 (*N* = 102)
Histology	DLBCL, tFL, PMBCL	DLBCL, tFL	DLBCL,PMBCL, FL, tFL
CAR T cell dosage	2 × 10^6^ cells/kg	3 × 10^8^ cells/kg	1 × 10^8^ cells/kg
ORR	83%	52%	75%
CR	58%	40%	55%
Median DOR (months)	11.1 (95% CI, 4.2—NE)	NR (95% CI, 10—NR)	NA
Overall survival	24-month survival, 50.5% (95% CI 40.2–59.7)	11.7 months (95% CI, 6.6—NE)	NA
Any grade CRS/NT	93%/64 %	58%/21%	37%/25 %
Grade ≥ 3 CRS	13%	22%	1%
Grade ≥ 3 NT	28%	12%	15%
Tocilizumab/steroid usage	43%/27%	15%/10%	17%/21%
Grade 5 AEs	4%	None	None
Reference	[11, 13]	[9, 14]	[12, 15]

*Abbreviations*: *N* number of patients, *ORR* overall response rate, *CR* complete response rate, *CRS* cytokine release syndrome, *NT* neurotoxicity, *DOR* duration of response, *CAR* chimeric antigen receptor, *AE* adverse event, *DLBCL* diffuse large B cell lymphoma, *tFL* transformed follicular lymphoma, *FL* follicular lymphoma, *PMBCL* primary mediastinal B cell lymphoma, *NE* not estimated, *NR* not reached, *NA* data unavailable

Currently, there are more than 200 clinical trials evaluating the role of CAR T cells in lymphoma. Severe toxicities including life-threatening cytokine release syndrome (CRS) and neurologic dysfunction vary according to the CAR T cell product. These toxicities occurred in the early phase clinical trials [9, 11] and require specialized management. The challenge remains in predicting patients who will have these toxicities and early recognition and management of these toxicities outside of a specialized center (or a large academic center). Financial toxicity related to pricing and reimbursement of CAR T cell therapy remains unresolved.

#### Redesigned CAR T cell therapy

Despite the excellent responses seen with CAR T cell therapy, the toxicities including CRS and neurotoxicity remain a challenge. Varying rates of grade 3 CRS and neurotoxicity have been reported in CAR T cell studies for r/r diffuse large B cell lymphoma (DLBCL) ranging from 13–14% CRS, 7–28% neurologic dysfunction, and two deaths from these toxicities [9, 11]. These are secondary to rapid in vivo T cell expansion, systemic perturbation of the immune system with release of inflammatory cytokines, and endothelial damage causing disruption of blood-cerebrospinal fluid barrier [16]. A novel approach to mitigate the risk for CRS has been to channel signaling via an endogenous CD-3 complex along with a redesigned T cell activating antigen receptor to regulate the cellular responses after activation. The ARTEMIS™ signaling platform has been coupled with Eureka’s human anti-CD-19 antibody, ET190L1, and this novel complex is expressed on primary T cells through genetic modification [17]. In vitro, the re-engineered complex has been able to retain the potency and has shown a significant reduction in cytokine release during antigen-specific T cell activation [17]. In comparison to CAR T cells, in-vitro studies of ARTEMIS™ T cells secreted less cytokines including interleukin (IL)-2, interferon- gamma (IFN-γ), granulocyte-monocyte colony stimulating factor (GM-CSF), and tumor necrosis factor alpha (TNF-α) [17]. They also demonstrated less propensity for T cell exhaustion compared to CAR T cells. The engineered T cells were given in first in human clinical studies and initial reports of 21 heavily pretreated r/r B cell lymphoma patients shows a favorable safety profile with no CRS or neurotoxicity reported [18]. At a median follow-up of 3 months (range 1–8 months), 21 patients completed the first month efficacy assessment with 52% overall response rate (ORR). Five of the six patients with complete response (CR) remained in CR at the end of 6-month assessment [19]. Plasma levels of cytokines IL-2, 4, 6, 8, 10, IFN-γ, and TNF-α and GM-CSF were below levels of detection post-treatment. Patients with r/r lymphomas have been treated at three different dose levels, with good response and no serious adverse events (SAE) leading to treatment discontinuation, CRS, or neurotoxicity. This novel T cell platform appears to have promising efficacy in r/r NHL with a favorable toxicity profile with no CRS and neurotoxicity seen.

#### Bispecific CAR T cells

Relapses and resistance to CAR T cell therapy may be secondary to antigen escape and low level of antigen expression in CD-19 positive and CD-22 positive tumors [20–22]. Targeting multiple antigens can minimize the risk of antigen escape and improve the on-tumor specific effect by CAR T cell therapy. The advantage of a bispecific CAR T cell stems from the probability of loss of two different antigen targets is low and the bispecific CAR T cell has improved avidity to dual antigen-positive cancer cells compared to a monospecific CAR T cell, in particular at low antigen densities. In a phase 1 study, a bispecific CAR T cell targeting CD-19 and CD-22 has been evaluated in seven patients of which five had DLBCL and two had ALL [23]. Among the patients with DLBCL, the ORR was 80% with a 40% CR. No grade 3 adverse events (AE) were reported; however, six patients developed reversible CRS and three patients developed neurotoxicity [23]. Given tolerable toxicity and good efficacy, a dose escalation and expansion study with 60 patients is planned. A different bispecific CAR T cell product targeting CD-19 and CD-20 has been evaluated in r/r NHL with mantle cell lymphoma (MCL), DLBCL, and chronic lymphocytic leukemia (CLL) in two escalating doses in a phase 1 study [24]. The ORR was 50% including 33% CR and no grade 3 neurotoxicity or CRS was reported. Two patients developed grade 1–2 CRS and neurotoxicity. No DLTs were reported.

A bispecific CAR T cell targeting CD-19 and CD-22 is currently being studied in a phase 1 study, and enrolled patients will receive 3 doses of consolidation therapy with the anti-programmed death (PD) 1 monoclonal antibody pembrolizumab [25]. Six patients with r/r DLBCL and two patients with transformed follicular lymphoma (tFL) and transformed marginal zone lymphoma were treated with this novel bispecific CAR T cell in escalating doses in a phase 1 study [25]. One patient developed grade 3 neurotoxicity which was reversible and one patient developed grade 2 CRS with other grade 3 toxicities listed in Table 2. Four out of five patients responded with an ORR of 80% and 40% CR. Escalation to higher doses and updated follow-up of patients is being planned.

**Table 2:** Cellular therapies

Cellular therapy	*N*	Patient population	ORR	CR	Grade 3 CRS/NT	Grade 3 AE	Ref
ET190L1-Artemis^TM^ Therapy	21	DLBCL, FL,MCL, SLL/CLL, splenic MZL	52%	24%	None	Lymphopenia, neutropenia, tremor, fever, rash	[19]
Bispecific CAR (CD-19/CD-20)	7	DLBCL, ALL	60% (DLBCL)	40% (DLBCL)	None	None	[23]
Bispecific CAR (CD-19/CD-20)	6	DLBCL, FL, MCL,CLL	50%	33%	None	None	[24]
Bispecific CAR (CD-19/CD-22)	6	DLBCL, tFL, tMZL	80%	40%	1 patient (20%)—NT	Neutropenia, thrombocytopenia, hypophosphatemia, neurotoxicity	[25]
Armored CAR T cells	25	DLBCL ,CLL, tFL, FL, WM	72%	57%	2 patients (8%)—NT	Neurotoxicity	[26]
ACTR 087	7	r/r NHL	50%	33%	None	Neutropenia, leukopenia	[27]
ACTR 707	6	DLBCL, FL	NA	50%	None	Febrile neutropenia	[28]

*Abbreviations*: *N* number of patients, *ORR* overall response rate, *CR* complete response rate, *CRS* cytokine release syndrome, *NT* neurotoxicity, *AE* adverse events, *DLBCL* diffuse large B cell lymphoma, *CLL* chronic leukemic leukemia, *tFL* transformed follicular lymphoma, *FL* follicular lymphoma, *WM* Waldenström’s macroglobulinemia, *MCL* mantle cell lymphoma, *tMZL* transformed marginal zone lymphoma, *ALL* acute lymphoblastic leukemia, *NHL* non-Hodgkin lymphoma, *MZL* marginal zone lymphoma, *NA* data unavailable

#### Armored CAR T cells

The weak activity of CD-19-specific CAR T cells in NHL relative to ALL has been attributed to lack of persistence and expansion of CAR T cells and the blockage of function by the immune-suppressive microenvironment. The anti-CD-19 (19-28z/4-1BBL) “armored” CAR T cells have been engineered with both CD-28 and 4-1BB co-stimulation for increased tumor removal, continued T cell proliferation, and persistence [29]. In comparison to second-generation 19-28z or 19-4-1BBz CAR T cells, they achieve greater proliferation, IL-2 secretion, and persistence [29]. In a phase 1 trial, 25 patients with r/r NHL including de novo DLBCL, CLL, tFL, follicular lymphoma (FL), Waldenström’s macroglobulinemia (WM), and Richter’s transformation received varying doses of “armored” CAR-T cells, including 16 patients at the highest dose level 4 [3 × 10^6^ CAR T cells/kg] [26]. Fifty-seven percent (12 of the 21 patients) achieved CR, and at a median follow-up of 93 days (range, 30–439 days), 11 of the 12 patients remain in CR. CAR T cells were detected beyond 160 days. Sixteen patients experienced grade 1–2 CRS (67%) and no patient experienced severe CRS. Neurotoxicity rates were lower with only two patients developing reversible grade 3 neurotoxicity (8%). The armored CAR T cells appear efficacious with tolerable toxicity profile and encouraging responses in NHL.

#### Antibody-coupled T cell therapy

The loss of targeted antigen is one of the causes for treatment failure with CAR T cell therapy [30]. Targeting more than one tumor antigen can mitigate this and has been proven in pre-clinical models. The antibody-coupled T cell receptor (ACTR) platform is a novel engineered T cell therapy, composed of an extracellular domain of CD-16 linked to CD-3 signaling and 4-1BB co-stimulatory domains, and this mediates anti-tumor activity in combination with tumor-targeted antibodies [31]. The T cell through the CD-16 ectodomain binds to the Fc receptor in the antibody attached to the tumor antigen by the Fab portion. The T cells are activated by antibodies bound to tumor antigen and cause T cell activation, proliferation, and cytotoxic attack of target cells. The same ACTR T cell can kill different types of cancer cells in the presence of the right targeting antibody. Various antibodies including rituximab and transtuzumab along with ACTR T cells have shown excellent responses and tumor cell cytotoxicity in preclinical models [32]. In a phase 1 study for r/r aggressive CD-20 positive NHL, seven patients received ACTR087 in combination with rituximab at the first dose level [27]. At this level, there were no SAE and other notable toxicities including CRS, neurotoxicities, or autoimmune syndromes were not seen. Cytopenias were the most common AE. Out of the six patients evaluable for response, two patients achieved CR and one had a partial response (PR). Further dose escalation continues with enrollment of patients at dose level 2.

Another product, ACTR707, has been designed with a modified ACTR construct containing a CD-28 costimulatory domain instead of a 4-1BB co-stimulatory domain. In a phase 1 study for r/r NHL, six patients have been enrolled at the first dose level [28]. No dose-limiting toxicities have been reported among the four evaluable patients and a 50% CR has been observed. No CRS, autoimmune AE, or severe neurotoxicity was observed.

### Antibody-based therapies

Antibody-based therapies target tumor cells selectively through specific receptors or a distinct antigen expressed by the tumor. The discovery of the anti-CD-20 antibody rituximab has had a dramatic impact in the management of B cell lymphomas and immune-mediated disorders. Several newer antibodies have been approved for management of other malignancies. Newer antibodies against different target antigens and antibody-drug conjugates, which have been developed by combining targeted antibody with chemotherapy, are listed in Table 3 and will be discussed below.

**Table 3:** Antibody-based therapy

Drug	Antigen target	Class	Patient population	*N*	ORR	CR	Grade 3 AE	Ref
Blinatumomab	CD-19, CD-3	Bispecific Ab	DLBCL, FL, MCL	35	69 % 80% (FL) 71% (MCL) 55% (DLBCL)	37% 40% (FL) 43% (MCL) 36% (DLBCL)	Leukopenia, neurologic event	[33]
Blinatumomab	CD-19, CD-3	Bispecific Ab	DLBCL	25	36%	16%	Leukopenia, thrombocytopenia, neurologic event	[34]
CD20-Tcb (RG6026)	CD-20, CD-3	Bispecific Ab	DLBCL,PMBCL, tFL, RT, FL	64	38%	24%	None	[35]
Mosunetuzumab	CD-20, CD-3	Bispecific Ab	DLBCL, tFL, FL	98	41%	27%	Anemia, neutropenia, hypophosphatemia	[36]
Adct-402 (loncastuximab tesirine)	CD-19	ADC	MCL, FL	30	80% (FL) 46.7% (MCL)	53.3% (FL) 26.7% (MCL)	Increased GGT, neutropenia, anemia	[37]
Adct-301 (camidanlumab tesirine)	CD-25	ADC	B cell NHL T cell NHL	22 17	31.3% 50%	18.8% 0%	Immune-related AE: dermatitis exfoliative, thyroiditis	[38]
Adct-301 (camidanlumab tesirine)	CD-25	ADC	HL	60	38%	24%	Increased GGT, ALT, AST, and ALP, maculopapular rash, anemia, and thrombocytopenia; immune-related AE: Guillain–Barré syndrome, thyroiditis	[39]
MT 3724	CD-20, Shiga-like toxin-I A1	Recombinant fusion protein Ab	DLBCL	24	12.5%	4%	None	[40]
Hu5F9-G4	CD-47	Ab	DLBCL, FL	22	50%	36%	Pulmonary embolism, ITP, anemia, thrombocytopenia, neutropenia, pyrexia, chills	[41]
DCDS0780A	CD-79b	ADC	DLBCL, FL, MCL, MZL	48	40%	14%	Neutropenia, thrombocytopenia, hypercalcemia	[42]
177 Lu-lilotomab satetraxetan	CD-37	Antibody-radionuclide conjugate	FL, MCL, MZL, SLL	74	61%	26%	Neutropenia, thrombocytopenia, infections	[43]

*Abbreviations*: *N* number of patients, *ORR* overall response rate, *CR* complete response rate, *AE* adverse events, *DLBCL* diffuse large B cell lymphoma, *CLL* chronic leukemic leukemia, *tFL* transformed follicular lymphoma, *FL* follicular lymphoma, *WM* Waldenström’s macroglobulinemia, *MZL* marginal zone lymphoma, *RT* Richter’s transformation, *MCL* mantle cell lymphoma, *PMBL* primary mediastinal B cell lymphoma, *ADC* antibody-drug conjugate, *GGT* gamma-glutamyl transferase, *ITP* immune thrombocytopenic purpura, *AE* adverse event, *ALT* alanine aminotransferase, *ALP* alkaline phosphatase, *Ab* antibody

#### Bispecific T cell antibodies

T cell bispecific antibodies have been engineered to redirect immune effector T cells to eliminate malignant B cells, as a newer strategy in lymphomas. This approach is promising due to the synergy and complementary mode of combining T cell-mediated cytotoxicity with antibody-dependent cellular cytotoxicity (ADCC) [44]. However, in clinical practice, it is limited by infusion reactions, CRS, central nervous system toxicity, shorter half-lives, and need for continuous infusions.

Blinatumomab is a T cell-engaging bispecific (TCB) antibody which simultaneously links CD-3 and CD-19 antigen. The dual binding is constructed from a CD-19 specific single-chain antibody derived from the variable domains of murine monoclonal antibody HD37 and an anti-CD3 portion derived from murine antibody L2K [45]. Using recombinant DNA technology, the two single-chain variable domain fragments are combined by a glycine-serine linker sequence for the production of TCB [46]. The cell lysis by blinatumomab occurs via multiple mechanisms including direct binding to CD-19 and activation of T cells secreting granzymes and perforin from the synapse between T cells and target B cells [47]. In both in vitro and in vivo models, there is strong evidence for cytotoxic activity against CD-19 positive B cells [48, 49]. A phase 1 trial with blinatumomab dosage starting at 5 μg/m^2^/day as a continuous infusion enrolled 76 patients with relapsed NHL in an initial dose escalation phase and this was followed by a dose expansion phase at the maximum tolerated dose (MTD) of 60 μg/m^2^/day [33]. There were no responses noted at doses ≤ 15 μg/m^2^/day, indicating a dose-response relationship. At the 60 μg/m^2^/day dose, the ORR was 69% and CR/complete remission unconfirmed (CRu) was 37% with long-term remissions noted independent of prior therapies and histologic subtype. Impressive single-agent activity was seen in follicular lymphoma (ORR 80%), mantle cell lymphoma (ORR 71%), and DLBCL (ORR 55%) [33]. Three grade 5 AEs were reported including two deaths related to infection. The most common grade 3 AE were lymphopenia at 69% and grade 3 neurologic events at 22% with encephalopathy (8%), headache (4%), and aphasia (4%) being the most common neurologic manifestations. Neurologic events began within the first 2 days of the first infusion and resolved with treatment or discontinuation. These events have been attributed to cytokine-releasing T cells migrating into the central nervous system (CNS). The therapy-related neurologic events caused frequent discontinuation, and several approaches to mitigate this are being explored including single-stepwise dose escalation with pentosane polysulfate SP54 or double-stepwise dose escalation with corticosteroid prophylaxis.

A phase 2 study in r/r DLBCL evaluated the safety and efficacy of blinatumomab and reviewed the optimal administration through either stepwise dose escalation to maximal target dose or treatment on a fixed target dose [34]. Twenty-five patients with r/r DLBCL were enrolled including 16 patients with refractory disease at baseline. The ORR was 36% with a CR of 16% and a median duration of response of 11.6 months. The response rate for refractory patients was lower (ORR 19%) in comparison to patients with relapsed disease (ORR 67%). Median PFS was 3.7 months (95% CI, 1.4–7.7) with median overall survival (OS) of 5 months (95% CI, 2.3 to not estimable). Grade 3 neurologic events reported were encephalopathy (9%) and aphasia (9%) with no patient experiencing grade 4 or 5 AEs. Two patients who received the flat target dose (112 μg/day) at therapy initiation developed serious grade 3 neurologic events related to therapy, and one patient developed grade 4 respiratory failure related to therapy and other grade 4 AEs unrelated to therapy including neutropenia and bone marrow toxicity from an acute viral infection were reported. Further enrollment was terminated in this cohort for safety reasons after review by the data monitoring committee. Currently, there are clinical trials evaluating the combination of blinatumomab with other immunomodulatory agents like lenalidomide (NCT02568553) and immune therapies like pembrolizumab (NCT03605589, NCT03340766) in relapsed and refractory lymphoma.

The CD-20-TCB (RG6026) is another TCB antibody designed to bind to CD-20 and CD-3 receptors in a “2:1” format, with high-avidity binding from two CD-20 binders and a CD-3 binder and strong potency enabled by a head-to-tail orientation and a long half-life [50, 51]. It has potent activity in primary tumor samples, and in vivo, it has shown regression of aggressive lymphoma models [52]. A single dose of obinutuzumab pretreatment has been shown to debulk the disease and abrogate the initial strong CRS associated with T cell activation [52]. In a phase-1, first in human trial, 47 patients with aggressive r/r B cell lymphomas and 17 patients with r/r indolent lymphomas received CD-20-TCB at doses ranging from 5 μg to 1800 μg in an every 2 weeks schedule [35]. The most common AEs included pyrexia, neutropenia, and grade 1–2 CRS in 14 patients. All CRS events were manageable with no central nervous system toxicity reported. CR was noted from 300 μg dose onwards after two cycles of therapy in 29 evaluable patients. Investigator-assessed ORR was 38% with 24% CR rate and all CRs were sustained at a median follow-up of 96 days (range 26–152).

Mosnetuzumab is a humanized, bispecific antibody that has been developed with activity binding to CD-3 epsilon (CD-3ε) expressed by T cells and CD-20 expressed in B cell lymphomas [53]. In in vitro and in vivo studies, it showed activity against normal and malignant B cells and it activated T cell-dependent (TDB) killing via the granzyme-perforin pathway. It also demonstrated anti-CD-20-TDB activity against cell lines with very low CD-20 expression levels and only a transient CRS was noted in the first 24 hour, despite the long half-life for this antibody. In a phase 1/1b study of 98 r/r NHL patients, mosunetuzumab was administered in escalating dose design in two different dosing strategies [36]. Sixty-six percent of the patients developed treatment-related AEs, and 22.5 % were grade 3 with the majority events occurring in cycle 1. Two treatment-related deaths including one secondary to hepatic failure and one from hemophagocytic lymphohistiocytosis from Epstein-Barr virus infection occurred. Responses were noted in patients refractory to prior anti-CD-20 and in patients relapsing after CD-19 targeted CAR T cell therapy and are listed in Table 3.

#### Antibody-drug conjugates

Antibody-drug conjugates (ADCs) are a novel class of drugs which consist of cytotoxic chemotherapy combined to a target-specific monoclonal antibody via a linker. These combine the cytotoxic potency of chemotherapy with the selectivity of monoclonal antibody to provide a novel safe and effective therapy. Several ADCs have been studied and are in current clinical practice including brentuximab vedotin, inotuzumab ozogamicin, and trastuzumab emtansine.

ADCT-402 is a CD-19-targeted antibody-drug conjugate (ADC) carrying SG3199 which causes cytotoxicity by DNA crosslinking [54]. It is strongly potent and selectively targets CD-19 expressing cell lines and is effective through bystander killing of CD-19 negative cells as well. In a phase 1, multicenter, open-label, single-arm study, with dose escalation and dose expansion cohorts of 137 r/r DLBCL patients, 15 to 200 μg/kg of ADCT- 402 was given for a median two cycles (range 1–13) [55]. The ORR was 40.2% in 132 evaluable patients with 22% achieving CR. At a median follow-up of 5.13 months, the median duration of response (DOR) was 4.17 months; although for those achieving CR, the median DOR has not been reached. The most common grade 3 AEs included elevated gamma-glutamyltransferase and cytopenias. ADCT-402 has shown good single-agent antitumor activity and the toxicity profile is manageable at doses ≥ 120 μg/kg.

CD-25 is expressed by many lymphomas including HL, peripheral T cell (PTL), cutaneous T cell (CTCL), and NHL [56]. ADCT-301 (camidanlumab tesirine [Cami-T]) is an ADC containing monoclonal antibody specific for CD-25 conjugated to a pyrrolobenzodiazepine dimer toxin. In vivo studies of ADCT-301 have demonstrated high potency and selective cytotoxicity against CD-25 expressing human lymphoma cell lines [57]. Once internalized, the dimer toxin causes cytotoxic effects through the formation of DNA interstrand cross-links. In a phase 1 study of 60 patients with heavily pretreated classical HL, dosing ranges from 5 to 300 μg/kg were evaluated [39]. The MTD was not reached; however, 45 μg/kg every 3 weeks was chosen for the dose expansion phase. Analysis of the 45 μg/kg dose group (dose escalation with expansion cohort) showed an ORR of 80.8% (21/26 pts) and CR rate of 50% (13/26 pts). The median PFS was 6.7 months and median DOR was 7.7 months. The most common grade 3 AEs noted were liver function abnormalities, anemia, thrombocytopenia, and a maculopapular rash. Grade 3 or higher AEs were seen in 37/60 (61.7%) patients resulting in treatment discontinuation in 17/60 (28 %) of the patients. Immune-related AEs were reported including two cases of Guillain–Barré syndrome (one each at dose 45 and 60 μg/kg) and one case of thyroiditis. Although encouraging ORRs were seen in this heavily pretreated HL population, cautious evaluation in further phase 2 studies of this novel ADC will be required given the immune-related AEs.

The ADCT-301 has been evaluated in a phase 1 trial of 39 patients with r/r NHL and T cell lymphomas [38]. Dosages evaluated in this population range from 3 to 150 μg/kg with a median number of 2 cycles (range 1–5) and a median treatment duration of 22 days (range 1–127). The grade 3 AE profile is similar to the previous study discussed with ADCT-301, and other immune-related AEs were reported in five patients. However, severe neurological impairment like Guillain–Barré syndrome was not seen. The MTD was not reached, but at doses of 60–150 μg/kg, the ORR was 38.5 % (10/26 pts) with 11.5% CR. The T cell lymphoma cohort had an ORR of 50 % (all PR), and enrollment in the 60 μg/kg and 80 μg/kg cohorts is ongoing to evaluate the optimal dose for further expansion in each subtype. The B cell lymphoma cohort treated at doses ≥ 60 μg/kg had a less impressive 31% ORR with 18.8% CR. This ADC appears to have promising activity in T cell lymphomas with a tolerable toxicity profile, and further dose evaluation is underway with planned dose expansion at the MTD.

#### Engineered toxin antibody

Engineered toxin body (ETB) is a novel recombinant therapy targeting cancer cells combining an immunotoxin scaffold with an antibody fragment binding domain. They are designed to create a targeted response based on antibody binding, intracellular internalization, and ribosomal inhibition by a Shiga-like toxin [58]. This unique delivery platform has been designed to avoid the innate and adaptive immune recognition. MT-3724 is an engineered toxin antibody (ETB) that comprises of a single-chain variable fragment of an antibody targeting CD-20 and a Shiga like toxin subunit A inactivating the ribosomal activity. In a first in human study with MT-3724, 24 patients with r/r NHL have been treated including 21 patients in 6 dose escalation doses (range from 5–100 μg/kg/dose) and three patients in the MTD cohort at 75 μg/kg/dose [40]. Peripheral edema, fatigue, diarrhea, myalgia, and cough were the most common AEs reported. In the dose expansion cohort, two out of three patients developed grade 2 capillary leak syndrome (CLS) leading to dose delay and reduction. The CLS was attributed to obesity and was reversible in all patients. The MTD was reduced to 50 μg/kg/dose and capped at 6000 μg/dose. Five DLBCL patients had clinical benefit at 5–75 μg/kg/dose with 1 CR and 2 PR (ORR 12.5%) and two patients with stable disease demonstrated significant tumor reduction (49% and 48% respectively).

#### Macrophage-mediated phagocytosis

Hu5F9-G4 is a humanized, monoclonal antibody with anti-CD-47 activity that selectively induces phagocytosis of tumor cells through macrophages by unmasking pro-phagocytic “eat me” signals [59]. Hu5F9-G4-mediated phagocytosis is augmented by targeted antibodies like rituximab, and in pre-clinical models of lymphoma, the synergistic and durable anti-tumor effects of the combination have been demonstrated [60]. Advani and colleagues reported a phase-1b study of Hu5F9-G4 and rituximab in 22 patients with r/r DLBCL and FL who were treated with Hu5F9-G4 at a priming dose of 1 mg/kg IV followed by escalating weekly maintenance doses of 10 to 30 mg/kg [41]. Most of the AEs reported were grade 1 and 2, with the most common being chills, anemia, headache, and infusion-related reactions. The ORR was 50% with 36 % CR. In the DLBCL cohort, ORR was 40 % with 33% CR, and in the FL patients, ORR was 71% with 43% CR. Among patients with a response, 10/11 patients (91%) had an ongoing response at the time of data cutoff.

### Immune checkpoint therapy

The discovery of immune checkpoints and development of monoclonal antibodies that regulate these has revolutionized the oncology field over the past decade. Tumor immunity involves a multi-step process of antigen presentation, lymphocyte activation, recruitment of lymphocytes to the tumor microenvironment, and, finally, tumor cell death. The T lymphocyte activation requires the T cell receptor engagement with MHC on antigen presenting cells and co-stimulation by CD-28 interacting with B7-1 (CD-80) or B7-2 (CD-86) ligand on malignant cells [61, 62]. Numerous inhibitory receptors have been discovered, which may disrupt these T cell and tumor cell interactions and can dampen the activation process. The cytotoxic T lymphocyte-associated antigen 4 (CTLA-4) found on T cells shares homology with CD-28 and modulates the co-stimulatory signaling by competing with activating ligands like CD-80 and CD-86, expressed by antigen presenting cells and thereby suppressing T cell activation. Similarly, the PD-1 receptor expressed on T cells binding to programmed death ligand 1 (PD-L1) expressed by tumor cells can downregulate the T cell response. The immune checkpoint inhibitors can interfere with these interactions and activate anti-tumor activity by augmenting T cell activation. Currently, checkpoint inhibitor therapies targeting one of these ligands have been approved by FDA for treatment of multiple malignancies.

PD-L1 and PD-L2 are expressed by various hematologic malignancies, and in particular, PD ligand expression has been associated with 9p23-24 gene amplification [63]. In Hodgkin Reed–Sternberg cells, the gene amplification causes PD-L1/PD-L2 ligand expression directly and indirectly, from increased JAK2 expression through the JAK-STAT signaling pathway leading to further enhancement of PD ligand expression [64]. The higher frequency of 9p24 gene alteration and increased PD ligand expression makes classical HL responsive to immune checkpoint therapy. Excellent response rates with durable responses have been demonstrated in numerous single-agent studies with nivolumab or pembrolizumab in relapsed and refractory HL [65, 66]. Both nivolumab and pembrolizumab have been approved by the FDA for treatment of relapsed and refractory classical HL. Although the ORR with PD-1 blockade in HL is high, the number of patients achieving CR is low (16–22%) and there has been progression noted after the initial response [65, 66]. To improve upon its activity and maintain longer response, combinations including other checkpoint inhibitors like ipilimumab, antibody drug conjugates like brentuximab or chemotherapy have been tested. In a phase 1 study, nivolumab in combination with ipilimumab showed response rates comparable with single-agent nivolumab, with increased toxicities [67]. There are ongoing trials with blockade of other immune checkpoints including lymphocyte-activation gene 3 (LAG-3) in combination with immune checkpoint inhibitors (NCT02061761 and NCT03598608). Immunotherapy in combination with chemotherapy regimens like adriamycin, vinblastine, and dacarbazine (AVD) and in combination with brentuximab has been well tolerated [68, 69]. However, the response rates seen with the combinations were comparable to the activity seen with the respective regimens without the addition of immune checkpoint therapy [68, 69] and have not been encouraging.

NHL, unlike HL, have 9p24.1 gene alterations infrequently but the exception to this includes primary mediastinal B cell lymphoma (PMBCL), which shares histologic and genetic characteristics with HL, including 9p24.1 amplification and translocation [63]. Similarly, 9p24.1 copy number gains and translocations have been identified in primary central nervous system lymphoma (PCNSL) and primary testicular lymphomas (PTL), with rearrangement of the regulator elements of TBLX1XR1 leading to increased PD-L2 protein expression [70]. Thus, PMBCL, PCNSL, PTL, and gray zone lymphomas appear to share the genetic basis for immune checkpoint inhibition and suggest a potential role for PD-1 antibodies in these malignancies. In a phase 1 b study with pembrolizumab in 18 patients with r/r PMBCL, the ORR was 41% with 2 patients achieving CR [71]. There is an ongoing international phase 2 study confirming the efficacy of pembrolizumab in PMBCL and assessing if genetic abnormalities correlate with response (NCT 02576990). A case series of four patients with r/r PCNSL and CNS relapse of PTL treated with nivolumab off trial [72] included 100% ORR after 4 cycles, and at 17 months follow-up, all patients were alive.

The 9p24.1 genetic modification and rearrangements are rare in other lymphomas and PD-L1 expression is poor in aggressive B cell lymphomas [73, 74]. In a phase 1 trial of r/r DLBCL patients, nivolumab showed an ORR of 36%, but the responders had remission less than 3 months [75]. There are a few trials in DLBCL being completed with immune checkpoint inhibitors in combination with anti-CD-20 antibodies (NCT03401853) and immunomodulators and targeted agents like lenalidomide (NCT03015896) and copanlisib (NCT03484819). Follicular lymphomas (FL) do not express PD-L1 ligands or have chromosome 9 modifications, but immune checkpoint expression is prevalent on the TILs or other cells within the tumor microenvironment [76]. The PD-1 expression on the TILs appears to have an effect on progression and transformation risk in FL [77, 78]. Although FL exhibits some responsiveness to immune checkpoint therapy, the responses are significantly lower than seen in HL. Immune checkpoint antibodies in combination with anti-CD-20 antibodies like rituximab and obinutuzumab have demonstrated good tolerability and ORR ranging from 57 to 80% [75, 79] noted in various studies. These responses are comparable to historical controls treated with anti-CD-20 antibodies alone. There are ongoing trials evaluating checkpoint agents in combination with HDAC inhibitor (NCT03179930), radiation (NCT02677155), chemoimmunotherapy (NCT02541565), or personalized tumor vaccine (NCT03121677) in follicular lymphoma.

## Small molecule inhibitors

With recent advances, oncogenic mutations and dysregulation of signaling pathways have been identified as leading to lymphomagenesis and could be potential targets for therapy. We can selectively target these pathways and the molecules that are activated in lymphoma and known to be contributory to the survival of lymphoma cells. We will discuss some promising novel small molecules developed for different subtypes of lymphomas (Table 4).

**Table 4:** Small molecule inhibitors

Drug	Class	Patient population	*N*	ORR	CR	Grade 3 AE	Reference
M7583	BTK inhibitor	R/R DLBCL, WM, MCL, MZL, SLL	18	50%	11%	Diarrhea	[80]
ME-401	PI3Kδ	R/R FL, CLL	31	83%	NA	Diarrhea, rash	[81]
LAM-002A	Endosomal protein inhibitor	R/R DLBCL, MCL, FL, MZL, CLL	24	NA	NA	Tumor lysis syndrome	[82]
INCB057643	BET protein inhibitor	R/R FL, DLBCL	5	33% (DE)	None	Hyperglycemia, thrombocytopenia, anemia, hyperbilirubinemia, Increased INR	[83]
MRG-106	miR-155 inhibitor	Mycosis fungoides	38	NA	NA	Pruritus, tumor flare	[84]
DS-3201b	EZH1/2 dual inhibitor	PTL, AITL, DLBCL, FL, MZL	15	53%	6.6%	Pneumonia	[85]
Apatinib	VEGFR-2 tyrosine kinase inhibitor	R/R NHL	21	47.6%	9.5%	None	[86]

*Abbreviations*: *N* number of patients, *ORR* overall response rate, *CR* complete response rate, *AE* adverse events, *R/R* relapsed and refractory, *DLBCL* diffuse large B cell lymphoma, *CLL* chronic leukemic leukemia, *tFL* transformed follicular lymphoma, *FL* follicular lymphoma, *WM* Waldenström’s macroglobulinemia, *MZL* marginal zone lymphoma, *MCL* mantle cell lymphoma, *PTL* peripheral T cell lymphoma, *AITL* angio-immunoblastic T cell lymphoma, *NHL* non-Hodgkin lymphoma, *VEGFR-2* vascular endothelial growth factor receptor-2 tyrosine kinase inhibitor, *PI3Kδ* phosphatidylinositol 3 kinase delta, *BET* bromodomain and extraterminal protein inhibitor, *EZH* enhancer of zeste homolog, *miR* micro RN, *DE* dose escalation phase, *NA* data unavailable

### BTK inhibitor

The Bruton’s tyrosine kinase (BTK) enzyme is a regulator of B cell receptor-mediated signaling, and BTK inhibitors can effectively block several B cell functions and proliferation. BTK inhibitors like ibrutinib and acalabrutinib have been approved for B cell malignancies like CLL, MCL, marginal zone lymphoma (MZL), and WM. M7583 is a highly potent and selective second generation BTK inhibitor. In pre-clinical studies, it has shown selective kinase inhibition in comparison to ibrutinib and it does not inhibit the ADCC effects of rituximab in cell lysis studies [87]. In a phase 1 trial with M7583, 18 patients with r/r NHL have been enrolled at 5 different dose levels, including 900 mg daily the highest dose level [80]. The ORR was 50% with a disease control rate of 78% and two patients achieving CR. Two patients were reported to have treatment-related serious TEAE (treatment-emergent adverse event), and diarrhea was the most common TEAE in six patients (33%). The MTD has not been reached with no dose-limiting toxicities reported. Responses were observed at all doses and both 300 mg twice daily and 900 mg daily were recommended as optimal biological doses to be evaluated in the dose-expansion phase.

### PI3K inhibitor

ME 401 is a selective inhibitor of phosphatidylinositol 3 kinase p110 delta (PI3Kδ) expressed in B cell malignancies. PI3Kδ is responsible for the homeostasis and function of B cells and is involved in interacting with the tumor microenvironment. In a first in human study with ME-401, 31 patients with r/r FL and CLL were enrolled to receive escalating doses ranging from 60 mg daily to 180 mg daily [81]. The ORR was 83% including 75% in the FL patients and 100% in the CLL patients, with responses seen by cycle 2 in 20 out of 24 responding patients. Most common grade ≥ 3 AEs reported were diarrhea in 16%, rash in 10%, colitis in 6%, and stomatitis in 1%, all occurring in cycle 3 or later. There were no DLTs reported and no further dose escalation above 180 mg was planned. It was noted that the grade ≥ 3 AEs had delayed onset after cycle 2 and were reversible with drug interruption and steroid use and attributed to regulatory T cell suppression. Among the 31 patients, 18 patients were switched to intermittent dosing schedule, with the drug being administered on days 1–7 of a 28-day cycle if they had not experienced grade ≥ 3 AEs on continuous daily schedule [88]. Three patients (16%) among the 18 patients developed grade 3 diarrhea in cycles 1 and 2 of the intermittent schedule and have been re-treated with no recurrence of symptoms. The ORR was 90% among 30 evaluable patients. Another cohort of patients with relapsed FL, DLBCL, MZL, and MCL were enrolled with continuous dosing schedule for 2 weeks combined with rituximab and switched to intermittent schedule [88]. Ten out of the fifteen patients in this second cohort completed two cycles of continuous dosing and were switched to intermittent dosing. Only one patient out of 10 developed delayed grade 3 diarrhea and ORR was 70% (7/10) in patients with FL/MZL [88]. Thus, intermittent scheduled dosing for patients developing AEs had a lower rate of toxicity with similar efficacy.

### BET inhibitor

Bromodomain and extraterminal protein inhibitors can suppress B cell malignancies through epigenetic interactions causing proliferative pathway downregulation. INCB057643 is a selective small-molecule BET inhibitor. In a phase 1 study with advanced stage malignancies including lymphoma, a total of five lymphoma patients were enrolled [83]. Only one patient developed treatment-related AE in the form of thrombocytopenia. Among the lymphoma patients available for efficacy evaluation in the dose escalation cohort, one achieved CR and two had stable disease.

### Autophagy enhancers

Autophagy plays a vital role in cancer cell survival. Phosphatidylinositol-3-phosphate 5-kinase (PIKfyve) lipid kinase is an endosomal protein that regulates endolysosomal membrane transport and influences autophagy, by exposing damaged proteins to auto-phagolysosomes. LAM-002A has been identified as an inhibitor of PIKfyve and is cytotoxic in B cell lymphomas. This molecule disrupts lysosomal homeostasis, resulting in cytotoxicity with significant in vitro and in vivo antitumor activity in lymphoma models [89]. In a first in human study of 24 patients with r/r B cell malignancies including DLBCL, tFL, MZL, MCL, FL, and CLL, sequential cohorts of patients received various dosing of LAM-002A from 50 mg twice daily to 150 mg twice daily [82]. Dose levels up to 100 mg bid were well tolerated; however, at dose level 75 mg twice daily, nausea and vomiting led to drug discontinuation in two out of four patients. At a dose of 150 mg twice daily, SAEs including nausea and diarrhea occurred in 4 out of 4 patients with 3 patients discontinuing therapy and one patient requiring dose reduction due to diarrhea. There were no dose-limiting toxicities at the 125 mg twice daily dose and this was identified as the MTD. Enrollment is ongoing in a dose expansion at this dose level. At this dose level, one patient with DLBCL developed grade 4 tumor lysis syndrome. There have been partial metabolic responses noted in three patients with DLBCL treated at various doses (100 mg, 75 mg, and 125 mg). With the favorable toxicity profile and anti-tumor activity seen, further evaluation as monotherapy or in combination with chemoimmunotherapy is being considered.

### Micro-RNA

MicroRNA miR-155 is overexpressed in cutaneous T cell lymphomas like Sezary syndrome and mycosis fungoides. MRG-106 is an inhibitor of miR-155 and has been evaluated in a phase 1 trial as an intralesional, subcutaneous (SC), or intravenous (IV) rapid bolus or 2-h infusion [84]. Thirty-eight patients received IV or SC treatments with no SAEs attributed to MRG-106 at 22 months on study. Twenty-nine out of the 32 patients had improvement in modified Severity Weighted Assessment Tool (mSWAT) with 11 of the 21 patients receiving more than 1 month of therapy achieving greater than 50% reduction in mSWAT score. The reduction in mSWAT scores correlated with improvement in the quality of life measured by the Skindex-29 total score. MRG-106 has an acceptable toxicity profile, with clinical activity and encouraging improvement in quality of life.

## Conclusion

Relapsed and refractory lymphoma management remains a major treatment challenge. Although the addition of rituximab improved outcomes in patients with B cell lymphomas, a significant number of patients are rituximab refractory at the time of relapse. The development and approval of newer therapies including cellular therapy in the form of CAR T cells, the immunomodulator lenalidomide, the antibody-drug conjugate brentuximab, and the BTK inhibitor ibrutinib have further improved the outcomes of patients with relapsed disease in the last decade. Despite incorporating these agents into treatment at relapse, many patients will have poor outcomes at relapse with some unable to tolerate these therapies due to toxicity. The novel approaches described above have been designed to avoid the toxicities seen in current treatment options while some are targeting the disease by an entirely different approach. The promising efficacy demonstrated by these new treatments requires further evaluation in phase 2 or phase 3 trials. These therapies may ultimately enhance the efficacy of standard treatment options and further evaluation in combination approaches will be needed.

## Data Availability

Attached data
