# Correction to: Novel and emerging therapies for B cell lymphoma

**DOI:** 10.1186/s13045-020-01022-w

**Published:** 2021-02-16

**Authors:** Sabarish Ayyappan, Kimi Maddocks

**Affiliations:** Division of Hematology, Department of Internal Medicine, Arthur G. James Comprehensive Cancer Center, The Ohio State University Wexner Medical Center, 320 W 10th Street, A342 Starling Loving Hall, Columbus, Ohio 43210 USA

## Correction to: Journal of Hematology & Oncology (2019) 12:82 10.1186/s13045-019-0752-3

There is an error in Table 1 of the original article. Lisocabtagene maraleucel (liso-cel) is administered as a flat dose of 1 × 10^8^ CAR T cells, regardless of a patient’s weight. The table incorrectly lists the dose as 1 × 10^8^ cells/kg.

## References

[1] Ayyappan S, Maddocks K (2019). Novel and emerging therapies for B cell lymphoma. J Hematol Oncol..

